# Unique Properties of Yeast Probiotic Saccharomyces boulardii CNCM I-745: A Narrative Review

**DOI:** 10.7759/cureus.46314

**Published:** 2023-10-01

**Authors:** Sarath Gopalan, Sridhar Ganapathy, Monjori Mitra, Neha, Devesh Kumar Joshi, Krishna C Veligandla, Rahul Rathod, Bhavesh P Kotak

**Affiliations:** 1 Pediatrics, Madhukar Rainbow Children's Hospital, New Delhi, IND; 2 Pediatrics, Janani Children's Hospital, Mumbai, IND; 3 Pediatrics, Institute of Child Health (ICH), Kolkata, IND; 4 Medical Affairs, Dr. Reddy's Laboratories Ltd., Hyderabad, IND; 5 Medical Affairs, Dr. Reddy’s Laboratories Ltd., Hyderabad, IND; 6 Ideation and Clinical Research/Medical Affairs, Dr. Reddy’s Laboratories Ltd., Hyderabad, IND

**Keywords:** cncm i-745, paediatric acute gastroenteritis, antibiotic-associated diarrhoea, saccharomyces boulardii, probiotic

## Abstract

Probiotics, both bacterial and yeast, have long been associated with a beneficial health history and human well-being. Among yeasts, *Saccharomyces* is a genus that is efficacious in rendering better human health, with *Saccharomyces boulardii *(*S. boulardii*) CNCM I-745 being classified as a probiotic agent. The present review highlights the unique properties of *S. boulardii *and its rolein the prevention of antibiotic-associated diarrhea (AAD) and pediatric acute gastroenteritis (PAGE) in comparison to bacterial probiotics. Its unique properties,such as viability over a wide pH range, inability to acquire antibiotic resistance genes, and property to achieve a steady state rapidly, have given *S. boulardii* an edge over bacterial probiotics. In AAD patients, prophylactic use of *S. boulardii* has shown a significantly lower risk of AAD (in comparison to controls) and restored the diversity of gut microbiota. Among Indian children with PAGE,* S. boulardii *CNCM I-745 was found superior to *Lactobacillus rhamnosus *GG and four strains of *Bacillus clausii *in shortening the duration of diarrhea and reducing the length of hospital stay. *S. boulardii *CNCM I-745 being considered a safe probiotic for use in children and adults also finds recommendations in several international guidelines for the management of acute diarrhea. The current review discusses evidence for the proven efficacy and safety of *S. boulardii* CNCM I-745 as a probiotic for preventing gastrointestinal disorders.

## Introduction and background

The gut microbiome is invoked as a contributor to gastrointestinal (GI) ailments and a broad range of chronic human conditions, including cancer and diseases with inflammatory, metabolic, cardiovascular, autoimmune, neurologic, and psychiatric components [1,2]. Microbial dysbiosis is an imbalance of the structure and function of the gut intestinal microbiota, which is quite common in today’s World. Changes in diet, bacterial infections, and indiscriminate use of antibiotics are the common causes of dysbiosis [3].

**Figure 1:**
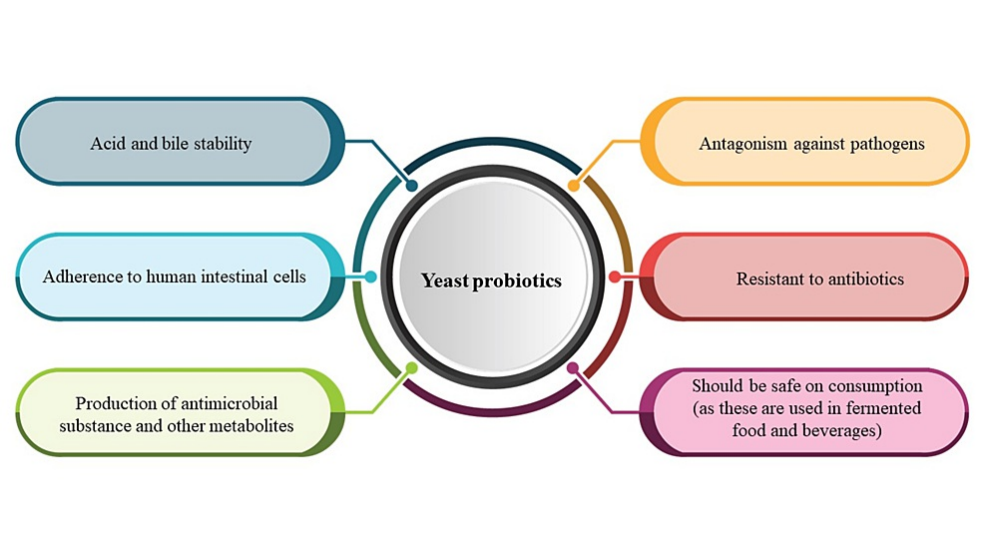
Advantages of yeasts as probiotics Adapted from Shruthi et al., 2022 [6] (CC BY-NC-ND license (http://creativecommons.org/licenses/by-nc-nd/4.0/))

One of the most efficient ways to restore gut microbial balance is probiotics [3]. Probiotic is a Greek term meaning “for life”. It is defined by the Food and Agriculture Organization of the United Nations (FAO) and the World Health Organization (WHO) as "live microorganisms that, when administered in adequate amounts, confer a health benefit on the host" [4,5]. There has been abundant research with probiotics of bacterial origin but limited work with probiotics of yeast origin. One of the rising threats of concern with probiotics of bacterial origin is their capability to transfer the resistant gene to pathogenic bacteria. This is where yeasts, which are naturally resistant to antibacterial agents, play a useful and important role as probiotics [6]. The other advantages of probiotics of yeast origin are demonstrated in Figure 1.

The yeast *S. boulardii* CNCM I-745 was the first yeast probiotic that was studied for the management of clinical disorders in humans. The *S. boulardii *strain is stable over a wide range of temperature levels and pH (including acidic conditions). It does not promote antibiotic resistance and has a beneficial effect against infections caused by pathogenic bacteria (e.g., *Clostridium difficile, Salmonella, Shigella, Escherichia coli*), viruses, and yeasts (mainly *Candida albicans*). Evidence supports the use of *S. boulardii* CNCM I-745 for treating several diseases [7].

The present review summarizes the current evidence on the role of *S. boulardii *as a biotherapeutic agent for the prevention of various GI diseases like antibiotic-associated diarrhea (AAD) and gut dysbiosis.

## Review

PubMed and NCBI databases were used for the search of the articles using the following keywords: probiotics, yeast as probiotics, and advantages of probiotics.

Open-access articles discussing the advantages of various yeast strains as probiotics were included in the review.

History of probiotics

Elie Metchnikoff, a Nobel laureate, was the first to report the beneficial effect of probiotics on human health. He suggested that lactobacilli may be considered a probiotic with a positive impact on health and the prevention of aging [4].

Beneficial effects of probiotics on human health

**Figure 2:**
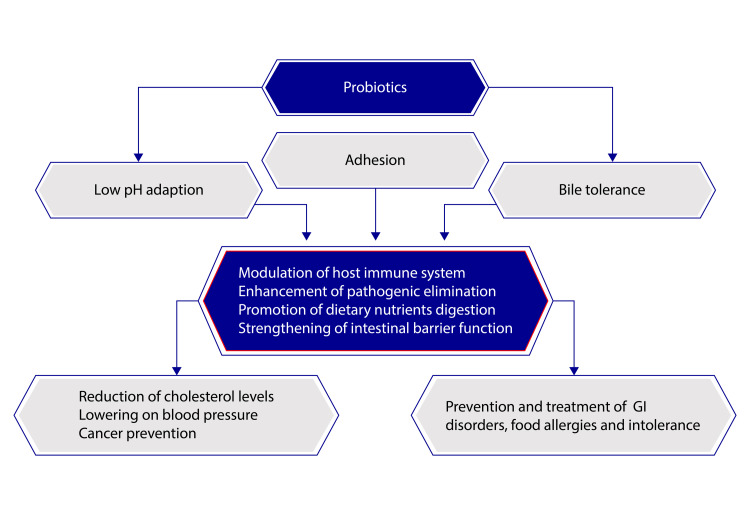
Some beneficial effects of probiotics on human health Adapted from Celebioglu et al., 2018 [8]. (Creative Commons Attribution (CC BY) license (http://creativecommons.org/licenses/by/4.0/)) GI: Gastrointestinal

Some beneficial effects of probiotics on human health are mentioned in Figure 2.

Sources of probiotics

Probiotic bacteria have been obtained from a variety of sources, including human breast milk, diets containing both plants and meat, human and animal feces, and animal guts [9]. Another source of probiotics is the human GI tract. From this source, several of the probiotic strains such as *Lactobacillus gasseri* and *L. reuteri* used today have been isolated. Besides, many animal species, including pigs, rats, and even poultry, have intestines that are rich in probiotics [10]. Also, several bacterial, fungal, and archaeal species are abundantly found in the human microbiome [11].

Properties of an ideal probiotic

The probiotic strains act differently against different pathogens by various actions. They act by directly killing or inhibiting specific pathogens, destroying pathogenic toxins, and reinforcing the host cell integrity. They also prevent the attachment of pathogen-host cells, restore the normal microflora, and balance the immune response (up or down-regulation).

All probiotics do not possess all of these mechanisms, but probiotics, such as *S. boulardii* CNCM I-745, are found to possess multiple anti-pathogen properties [12,13]. *S. boulardii* possesses several mechanisms of action (Figure 2) which can be divided into three main types, namely, luminal action, trophic action, and mucosal-anti-inflammatory signaling effects. *S. boulardii* in the intestinal lumen, might aid in interfering with pathogenic toxins and their attachment, interacting with normal microbiota, preserving cellular physiology, or re-establishing short-chain fatty acid levels. In addition, S. boulardii may also regulate the immune system, both within the lumen and systemically [14].

Yeast as a probiotic

Yeast probiotics with some unique properties have an edge over bacterial probiotics and the differences between them are listed in Table 1.

**Table 1:** Difference between bacteria and yeast and its implication on probiotics PPM: Phosphopeptidomannan; PLM: Phospholipomannan; LPS: Lipopolysaccharide; LTA: Lipoteichoic acid

	Yeast	Bacteria
Cell size [15]	10 µm	1 µm
Cell wall [15]	Chitin, mannose (PPM, PLM), glucan	Peptidoglycan, LPS (Gram-negative), LTA (Gram-positive)
Optimal growth conditions-pH [15]	4.5–6.5	6.5–7.5
Temperature (^0^C) [15]	20–30	10–80
Resistance to antibiotics [15]	Yes	No
Transmission of genetic material [15]	No	Yes
Autoaggregation [16]	Yes	Limited

Possible implications of the difference between yeast and bacterial probiotic properties

The difference between yeast and bacterial probiotic properties and their possible implications are mentioned in Table 2.

**Table 2:** Possible implications of the difference between yeast and bacterial probiotic properties GI: Gastrointestinal

Properties	Comments
Steric hindrance [16]	Yeast cells, due to their ~10 times larger size than bacteria, provide a steric hindrance against pathogenic bacteria. It increases the prospect of yeast being a probiotic agent
Immune response [17]	The cells of yeast contain many immunomodulatory components The outer layer of the cell wall consists of Mannoproteins that bind dendritic cell-specific intercellular adhesion molecule-3-grabbing non-integrin (DC-SIGN), Toll-like receptor 4 (TLR4), and others The middle layer contains β-glucans which bind Dectin-1 and TLRs 2 and 6 The innermost wall layer contains chitin, which binds the mannose receptor
Different sites of action in the GI tract [15]	Yeast is found both in the stomach and colon, suggesting their ability to survive in such different conditions (resistance to pH variation, tolerance of stress). This suggests that yeast is a good candidate as a probiotic because probiotics entering the GI tract must be resistant to variations in the pH
Resistance to antibiotics [15]	Bacteria can transfer the resistance genes to pathogenic bacteria, leading to antibiotic resistance transfer of genetic material which is not possible between bacteria and yeast, making yeast a potential candidate as a probiotic for the prevention of antibiotic resistance
Autoaggregation[16]	The ability of yeast strains to self-aggregate and produce flocs is the autoaggregation property This is a survival response in the presence of adverse environmental conditions like the human GI tract This provides a competitive advantage to the yeast cell over other microorganisms, including enteric bacteria Because, as compared to the bacteria, the yeast cells are larger and heavier, they sediment faster and in a larger amount

Properties of *S. boulardii* CNCM I‑745

**Figure 3:**
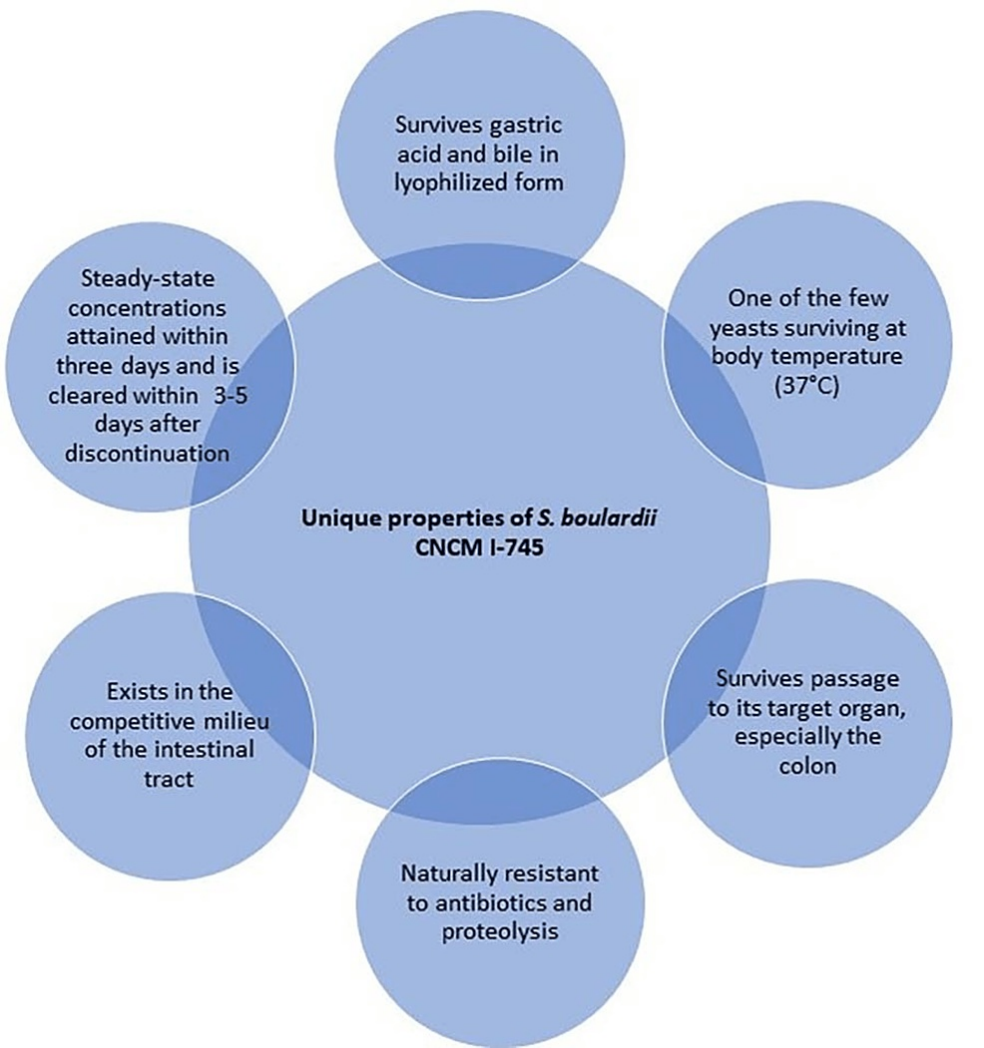
Unique properties of S. boulardii CNCM I-745 Image Credits: All authors

*S. boulardii* CNCM I-745 is a probiotic yeast of choice for the management of AAD and pediatric acute gastroenteritis (PAGE). Henri Boulard (French microbiologist) discovered *S. boulardii *CNCM I-745 in 1923, and it belongs to the *S. cerevisiae* species [18]. Due to its ability to produce different bioactive compounds, *S. boulardii* has taken a key position in the scientific community and is recently being used for managing gut diseases. Clinical evidence suggests that oral administration of *S. boulardii *is beneficial in managing multiple GI diseases including traveler’s diarrhea, AAD, C. difficile-associated syndrome, irritable bowel syndrome, and Crohn’s disease [19]. Due to the excessive use of antimicrobials, a continuous rise in multidrug-resistant organisms is reported. Therefore, in managing antibiotic resistance, probiotics, especially *S. boulardii*, can be used as an adjunct to the management of infections [19]. The unique properties of *S. boulardii *are mentioned in Figure 3 [20,21].

Mechanism of action of *S. boulardii *as a probiotic

**Figure 4:**
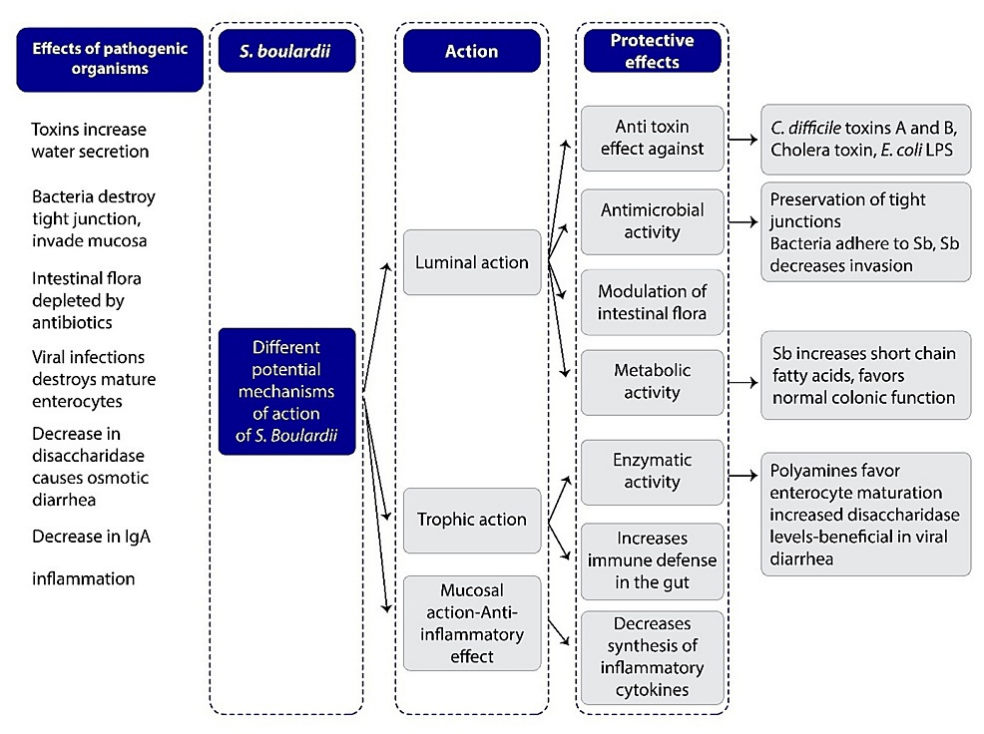
Different potential mechanisms of action of Saccharomyces boulardii Image Credits: All authors On the left various effects of different pathogenic microbes are shown. On the right, seven different protective effects of *S. boulardii* are mentioned. Within the lumen of the intestine, *S. boulardii* may degrade toxins of pathogens, interfere with pathogenic adherence, modulate normal microbiota, and preserve normal intestinal physiology. *S. boulardii* may also indirectly restore normal SCFA balance and increase secretory IgA levels or act as an immune regulator by influencing cytokine levels. LPS: Lipopolysaccharide; Sb: *S. boulardii*.

The gut microbiome performs a variety of functions, such as preventing pathogen colonization, maintaining the epithelial barrier, and controlling immune response [22]. *S. boulardii* similarly as a part of its probiotic effect has various modes of action like immunological and anti-toxin effects, modulation of intestinal flora, and impact on enzyme activity (Figure 4) [22].

Clinical benefits of *S. boulardii *as a probiotic in AAD and PAGE

Clinical evidence demonstrating the role of *S. boulardii* in the management of AAD and PAGE is given in Table 3.

**Table 3:** S. boulardii in the management of AAD and PAGE AAD: Antibiotic-associated diarrhea; PAGE: Pediatric acute gastroenteritis

Study/design	Population	Intervention	Results
*S. boulardii *for the prevention of AAD			
McFarland et al./ Meta-analysis, 10 randomized controlled trials (RCTs) [14]	Adults on antibiotics (n = 1869)	*S. boulardii* (200 mg – 1000 mg /day)	A significant therapeutic efficacy of *S. boulardii* in preventing AAD was reported (RR = 0.47, 95% CI: 0.35-0.63, P<0.001)
McFarland et al./ Meta-analysis 22 trials (23 treatment arms) [23]	Children (n = 4155) on antibiotics	Twelve trials tested a single strain of probiotic and 10 trials tested a mixture of probiotic strains	Analysis among single strains trials (12 trials) showed that *S. boulardii* significantly reduced AAD (pooled RR = 0.43, 95% CI: 0.32-0.60). All the probiotics were safe
Jindal et al./ Randomised, open, parallel study [24]	Children (n = 300, age 6 months-12 years) with upper respiratory tract infection or urinary tract infection receiving antibiotics	Control group: Antibiotic test group: Antibiotic + *S. boulardii* [BASE] 250 mg B.D.	A significant reduction in the incidence of diarrhea was observed in patients who received *S. boulardii* (P<0.001)
Szajewska et al./ Systematic review with meta-analysis, 21 RCTs [25]	Children and adults (n = 4780) receiving antibiotics for any reason, including Helicobacter pylori eradication therapy	Control group: Antibiotic + placebo/no treatment Experimental group: Antibiotic + *S. boulardii* at any dose/ duration	Among patients treated with antibiotics, a reduced risk of AAD was observed in the *S. boulardii* group vs placebo or no treatment, from 18.7% to 8.5% S. boulardii reduced the risk of diarrhea in children from 20.9% to 8.8% and in adults from 17.4% to 8.2% In children, S. boulardii also reduced the risk of C. difficile-associated diarrhea
Yang et al./ Meta-analysis, 21 studies[26]	Children (n = 3534 patients) on antibiotics	*S. boulardii*	For the prevention of AAD, the *S. boulardii* group could significantly reduce the diarrhea rate vs the control group In a Meta-analysis of 7 studies (523 patients), the *S. boulardii* group demonstrated a significantly higher efficacy rate in preventing AAD vs the control group
*S. boulardii* for the management of PAGE			
Padayachee et al./ Systematic review = 10 studies; Meta-analysis, 5 RCTs[27]	Infants and children (n = 619, aged <16 years) with a diagnosis of acute gastroenteritis (≥3 unformed stools in the last 24 h and of ≤48 h duration)	*S. boulardii* (250-500 mg/day) vs other treatments	*S. boulardii* significantly reduced the duration of diarrhea compared with control (MD –0.57; 95% CI –0.83 to –0.30; n = 548 children; 5 studies) and had a statistically significant effect on stool frequency on days 1, 2, 3 and 4 (P = 0.001) At the end of day 7, all children had solid stools
Szajewska et al./ Systemic review and Meta-analysis, 29 RCTs[28]	Children (n = 4217, aged 1 month to 15 years)	*S. boulardii* vs placebo or no intervention	*S. boulardii* was reported to be efficacious at a daily dose <300 mg/d (5 RCTs, n = 873, mean difference (MD) −0.84 d; high heterogeneity I^2^ = 91%; or 500 mg/d (15 RCTs, n = 2248, MD −0.86 d; I^2^ = 58%); or >500 mg/d (1 RCT, n = 41, MD −2.76 d) For those treated with *S. boulardii* compared with the control group, reduced duration of hospitalization (8 RCTs, n = 999, MD −0.85 d, 95% CI −1.35 to −0.34; I^2^ = 91%) was reported On day 2 to day 7 of treatment, *S. boulardii* reduced the risk of diarrhea Adverse effects were comparable between the groups
Ragavan et al./ Retrospective analysis[29]	Indian children (n = 160, age: 0 to 18 years) with acute diarrhea	Oral rehydration solution and zinc with or without *S. boulardii* CNCM I-745 (250 mg twice daily)	In the S. boulardii group, the median duration of diarrhea post-treatment was significantly shorter (3 days) vs the non-* S. boulardii* group (4 days) In the *S. boulardii* group, a significant reduction in the frequency of stools was observed post-treatment (1.7 vs 2.5 in the non-* S. boulardii* group).
McFarland et al./ A systematic review (22 RCTs) and meta-analysis (17 RCTs)[30]	Treatment for Indian PAGE patients (n = 4059)	Five single-strain probiotics and 3 multi-strained mixtures (Strains studied include *S. boulardii* CNCM I-745, L. rhamnosus GG, B. clausii O/C, SIN, N/R, T, Bifilac (4 strains), B. clausii UBBC-07, L. casei DN114001, L. sporogenes, and 8 strain mixture	*S. boulardii* CNCM I-745 had the strongest effect on shortening the duration of diarrhea (standardized mean difference, –1.86 d; 95% CI, –2.8 to –0.9), *S. boulardii* and *L. rhamnosus* GG significantly reduced hospital stays (−1.8 and −1.1 d, respectively), while B. clausii had no effect* S. boulardii* significantly reduced the frequency of stools/day by day 4, and *L. rhamnosus* GG was reduced by day 5
Fu et al./ Systemic review and meta-analysis (10 studies)[31]	Children with acute gastroenteritis (n = 1282, aged <5 years)	*S. boulardi*i vs control	*S. boulardii* could effectively shorten diarrhea duration (MD = 19.70, 95% CI, -24.87, 14.52) and reduce the length of hospital stay (MD = −0.91, 95% CI: -1.28, -0.54) A significantly lower relative risk of continued diarrhea was observed in the treatment group after 1-day treatment (RR = −0.31, 95% CI, 0.59, 0.03) and 3 days of treatment (RR = 0.52, 95% CI: 0.41, 0.66) vs the control group After 3 days of treatment, S. boulardii reduced the average number of diarrhea by about 1.03 (MD= −1:03, 95% CI: -1.53, -0.53) No adverse drug reactions were reported in either group
*S. boulardii* vs other probiotics for management of AAD and PAGE			
McFarland et al./ meta-analysis[32]	Children on antibiotics	Sixteen different types of probiotics were combined	*S. boulardii* and *L. rhamnosus* displayed significant efficacy for pediatric AAD when pooled (pooled RR 0.43, 95% CI 0.21-0.86).
Vineeth et al.[33]	Indian children suffering from rotaviral diarrhea (n = 80)	The average dose of *S. boulardii *(500 mg/day) vs B. clausii (10 mL/day)	In the *S. boulardii* group, the total mean duration of diarrhea was significantly shorter vs the *B. clausii* group
Blaabjerg et al./ Systematic review and meta-analysis, 17 RCTs[34]	Outpatients of all ages on antibiotics (n = 3631)	Lactobacilli spp., Lactococcus spp., Streptococcus spp. Bifidobacterium spp., Saccharomyces spp., Leuconostoc cremoris, Bacillus spp., Clostridium spp., or alone or in combination	In a subgroup analysis, *S. boulardii* (four studies) showed a significantly lower risk of AAD vs control (RR 0.41; 95% CI 0.30 to 0.57), while this was not observed in the combination of L. acidophilus La-5 and B. lactis Bb-12 (2 studies) (RR 0.79; 95% CI 0.47 to 1.33)
Vidjeadevan et al./ RCT[35]	Children (n = 105, aged 6-36 months) with acute diarrhea	Group A received ORS and zinc; Group B received ORS, zinc and *S. boulardii*; Group C received ORS, zinc, and B. clausii	The mean duration of diarrhea was 108 hours for group A, while 72 hours and 96 hours for group B and group C, respectively. The mean duration of diarrhea was highest in group A (108 hours) and lowest in group B (72 hours)
Johnston et al./ Cochrane review, 33 studies[36]	Children receiving antibiotics (0 to 18 years, n = 6352 participants)	Probiotics assessed included Bacillus spp., Bifidobacterium spp., Clostridium butyricum, Lactobacilli spp., Lactococcus spp., Leuconostoc cremoris, Saccharomyces spp., or Streptococcus spp., alone or in combination, placebo or no treatment	Among various probiotics, placebo or no treatment, *L. rhamnosus* or *S. boulardii* at 5 to 40 billion colony forming units/day were the most appropriate probiotics for the prevention of AAD
Li et al./ Bayesian network Meta-analysis (21 interventions)[37]	Children with acute diarrhea (n = 13,443)	Probiotic interventions could be divided into single-strain and multi-strain probiotics. The single-strain probiotics included *S. boulardii, L. rhamnosus GG, L. reuteri, B. clausii, L. acidophilus, B. lactis, L. sporogenes, L. plantarum, E. coli Nissle *1917 (ECN 1917), *L. paracasei, and E. faecium.* Multiple-strain probiotics included L. species (spp.), L. spp. + B. spp., L. spp. + B. spp.+ S. spp., L. spp. + S. spp., B. spp. + S. spp., Bacillus spp. + E. spp. + Clostridium spp., L. spp. + B. spp. + E. spp., L. spp. + B. spp. + Pediococcus spp., and L. spp. + S. spp. + C. spp. + Bacillus spp. Control arm: Placebo/no treatment	*S. boulardii* among all the probiotics was the most effective in reducing both duration of diarrhea (vs placebo) and the risk of diarrhea lasting ≥2 days (vs placebo or no treatment). It [Odds ratio (OR) = 0.22; 95%CI, 0.11, 0.41] significantly decreased the risk of diarrhea lasting ≥2 days versus placebo or no treatment
Altcheh et al./A two-arm parallel, randomized trial[38]	Children (n = 317, 6 months to 5 years old) with mild-moderate acute diarrhea	Randomized to 5 days of either *S. boulardii* CNCM I-745 (n = 159) or a 4-strain mixture of B. clausii (n = 158). Post-probiotic treatment follow-up was done for 7 days	*S. boulardii* CNCM I-745 displayed a significant decrease (P = 0.04) in the mean duration of diarrhea (64.6 hours, 95% CI 56.5-72.8) in comparison with B. clausii (78.0 hours, 95% CI 69.9-86.1) Both treatments were well tolerated

Safety of *S. boulardii*

A recent study reports that the probiotic *E. coli* strain Nissle 1917 produces* in vitro* and *in vivo* colibactin and subsequently induces mutagenic DNA damage. This is a serious safety concern that should not be ignored keeping in mind the health of patients and the general public overall [39]. As the genotoxic activity of this strain cannot be dissociated from its probiotic activity, the safety aspects of large-scale use of this probiotic strain need to be reassessed. This brings up an important issue of side effects that may be associated with any effective probiotic [40].

*S. boulardii* CNCM I-745 is considered to be a safe probiotic. Clinical trials have not reported any side effects with *S. boulardii *[19]. Very rarely, in critically ill and/or immunocompromised patients, increased amounts of *S. cerevisiae* infections (fungemia) have been observed [41]. For immunocompromised patients, even opening a packet of *S. boulardii* can lead to air contamination, increasing the risk of infection [42]. However, meta-analysis has reported* S. boulardii* to be safe in children suffering from acute diarrhea [43].

Global recommendations for *S. boulardii* as a Probiotic

Table 4 provides the global recommendations for *S. boulardii *as a probiotic.

**Table 4:** Global recommendations for S. boulardii as a probiotic Abbreviations- IAP: Indian Academy of Paediatrics; ESPGHAN: European Society for Paediatric Gastroenterology; Hepatology, and Nutrition AAD: Antibiotic-associated diarrhea; AGE: Acute gastroenteritis; HP: Helicobacter pylori.

Sr. No.	Recommended Strain	Recommending agency	Year of recommendation	Recommended indication (dose)	Not recommended	Quality of evidence
1	S. boulardii	IAP[44]	2022	Adjuvant therapy in acute diarrhea (250-750 mg/day)		Very low-to-low
2	S. boulardii	ESPGHAN [45]	2022	Acute gastroenteritis in children (250–750 mg/day)	-	Low
				Prevention of AAD (≥5 billion CFU per day)	-	Moderate
	S. boulardii	ESPGHAN[46]	2020	Acute gastroenteritis in children as an adjunct treatment to oral rehydration therapy (250-750 mg/day)		Low
	Bacillus clausii			-	NOT recommended for the prevention of AAD	Very low
3	S. boulardii	ESPGHAN[47]	2016	Preventing AAD in children		Moderate
				Prevention of C. difficile-associated diarrhea in children		Low
4	S. boulardii CNCM I-745	World Gastroenterology Organization[48]	2017	AAD in adults (5x10^9^ CFU/capsule or 250 mg twice daily)		Level 1
				Prevention of C. difficile–associated diarrhea (or prevention of recurrence) in adults (5x10^9^ CFU/capsule or 250 mg twice daily)		Level 3
				Co-adjuvant therapy for HP eradication (5x10^9^ CFU/capsule or 250 mg twice daily)		Level 1
				Prevention of AAD (250–500 mg)		Level 1
5	S. boulardii CNCM I-745	Latin-American Experts[49]	2015	Acute infectious diarrhea		Grade of evidence:1a
				Prevention of AAD, prevention of traveler’s diarrhea		Grade of evidence:1b
6	S. boulardii	European Paediatric Association Expert Panel[50]	2018	Prevention of AAD, acute gastroenteritis (adjunct to the oral rehydration therapy) (250-750 mg), Prevent C. difficile-associated diarrhea		-
7	S. boulardii	World Journal of Gastroenterology-2017 Asia Pacific (APAC) region[51]	2017	For AGE, in adjunct to oral rehydration therapy		Strong
				AAD		Strong
				C. difficile-associated diarrhea		Low
8	S. boulardii	ESPGHAN [45]	2023	AAD		Moderate
				Acute gastroenteritis		Low

## Conclusions

Probiotic *S. boulardii* CNCM I-745, by its unique properties, acts in a variety of ways to exert its pathogen-binding, immunological, and anti-toxin actions. Additionally, it cannot transfer genetic material, making it a potential candidate as a probiotic for preventing antibiotic resistance. *S. boulardii* stands out among the other probiotics as one of the most effective for avoiding AAD in children. In comparison to* L. rhamnosus GG* and a few strains of *B. clausii*, *S. boulardii* CNCM I-745 shows a significantly reduced mean duration of diarrhea in PAGE patients. Furthermore, because of its proven efficacy and safety, it is recommended by ESPGHAN and other global bodies for the prevention and treatment of acute diarrhea. Thus, *S. boulardii *CNCM I-745 is one of the preferred choices of probiotics for the management of AAD and PAGE due to its distinct advantages over bacterial probiotics as well as its favorable efficacy and safety profile.
